# Influence of Temperature on the Chemical and Rheological Aging Kinetics of Corn Starch-Modified Bitumen

**DOI:** 10.3390/ma19153302

**Published:** 2026-08-04

**Authors:** Paulina Rozpędowska, Małgorzata Wójcik, Mateusz Golda, Agnieszka Woszuk, Lidia Bandura, Szymon Malinowski, Wojciech Franus

**Affiliations:** Faculty of Civil Engineering and Architecture, Lublin University of Technology, Nadbystrzycka 40, 20-618 Lublin, Poland; d625@pollub.edu.pl (P.R.); malgorzata.wojcik2@pollub.edu.pl (M.W.); mateuszgolda13@gmail.com (M.G.); a.woszuk@pollub.pl (A.W.); l.bandura@pollub.pl (L.B.); s.malinowski@pollub.pl (S.M.)

**Keywords:** bitumen, aging, kinetic, FTIR, MSCR, Arrhenius equation

## Abstract

**Highlights:**

- Aging kinetics of corn starch-modified bitumen were quantitatively determined.
- Aging temperature strongly affected chemical and rheological transformation rates.
- FTIR and MSCR enabled kinetic evaluation of bitumen thermo-oxidative aging.
- Activation energies revealed the temperature sensitivity of aging reactions.

**Abstract:**

The increasing demand for sustainable bitumen modifiers has stimulated interest in bio-based materials capable of improving binder performance while reducing environmental impact. This study investigates the influence of corn starch on the thermo-oxidative aging kinetics of paving-grade bitumen. Unmodified 50/70 bitumen and binders containing 4, 6 and 8 wt.% corn starch were subjected to laboratory aging at 100 °C and 140 °C for up to 120 h. The aging process was evaluated using dynamic viscosity measurements, FTIR spectroscopy and Multiple Stress Creep Recovery (MSCR) testing. The obtained results demonstrated that corn starch significantly affected both the rate and temperature dependence of aging. The effect of corn starch was strongly dependent on both the aging temperature and modifier dosage, indicating that starch does not uniformly inhibit all aging processes but rather modifies their kinetics in a process-specific manner. Increasing the aging temperature from 100 to 140 °C accelerated the viscosity growth by approximately 9–11 times, depending on the binder composition. The apparent rate constants for carbonyl formation ranged from 9 × 10^−6^ to 7 × 10^−5^ h^−1^ for the reference binder and from 1 × 10^−5^ to 5 × 10^−5^ h^−1^ for starch-modified binders. The calculated apparent activation energies varied between 1.31 and 67.6 kJ mol^−1^, confirming that starch altered the temperature sensitivity of oxidation and structural transformation reactions. Among the investigated formulation (4–8 wt.% corn starch), the binder containing 4 wt.% corn starch exhibited the most favorable balance between aging resistance and production cost. Overall, the results demonstrate that corn starch modifies rather than universally inhibits bitumen degradation, with the optimum performance depending on the investigated aging parameter, modifier dosage and aging temperature, with 4 wt.% providing the most favorable overall balance between chemical aging behavior, rheological performance and production cost.

## 1. Introduction

Bitumen is a material obtained from crude oil through the refining and distillation process. Its properties are closely related to the source of the crude oil and the processing technologies employed [1]. It is primarily used in pavement applications as a binder that binds mineral aggregates into a cohesive mixture, thereby creating an integral structure capable of efficiently transferring loads within the surface and base layers [2]. Chemically, bitumen is a complex multicomponent mixture of aliphatic and aromatic hydrocarbons forming a colloidal system. With advances analytical techniques, bitumen is currently considered to consist of four main fractions: asphaltenes, resins, aromatics, and saturates [3]. According to the most widely accepted theory, bitumen is regarded as a colloidal system in which asphaltenes constitute the dispersed phase, while maltenes form the continuous dispersing medium. To describe differences in the colloidal structure of bitumen and the associated rheological properties, three principal colloidal structures have been distinguished: gel, sol, and sol–gel [4]. The type of colloidal structure depends on temperature as well as on the chemical composition and molecular structure of the binder. Studies have shown that the colloidal structure is influenced more strongly by the resin content and the chemical nature of maltenes than by the asphaltene content itself [5,6]. Asphalt pavement undergoes continuous aging throughout its service life as a result of exposure to various environmental and processing factors, including temperature, oxygen, moisture and ultraviolet (UV) radiation. This process causes irreversible changes in the chemical composition and colloidal structure of bitumen, ultimately leading to pavement deterioration [7,8]. Aging can be divided into two consecutive stages: short-term aging and long-term aging. Short-term aging occurs during the mixing, transportation, and placement of asphalt mixtures. At this stage, the binder undergoes oxidation and the evaporation of lighter oily fractions. In contrast, long-term aging progresses gradually throughout the service life of the pavement. During in-service aging, the asphalt binder is exposed not only to temperature and oxygen but also to UV radiation, precipitation, and de-icing agents [9,10]. Bitumen aging results in increased stiffness and brittleness of the binder, accompanied by a reduction in its adhesion to aggregates. Therefore, the performance of asphalt mixtures deteriorates, promoting the formation of pavement distresses, particularly cracking in the wearing course. These phenomena are primarily associated with the oxidation of bitumen constituents and the reduced resistance of the binder to moisture and freeze–thaw action [11,12].

Due to traffic loading and fluctuations in ambient temperature, pavements are susceptible to damage caused by permanent deformation, fatigue cracking, and low-temperature cracking. To address these challenges, an effective technique known as “bitumen modification” is widely employed. This approach involves the incorporation of one or more modifiers into bitumen to improve its physicochemical and viscoelastic properties, thereby enhancing pavement performance and extending its service life [2,13]. The most commonly used bitumen modifiers are polymers such as styrene–butadiene–styrene (SBS), polypropylene (PP), and polyethylene (PE). However, the relatively high cost of synthetic polymer-based modifiers has created a need for the development of less expensive alternative materials [14,15]. In recent years, increasing attention has been paid to the use of bio-based materials capable of partially or completely replacing conventional petroleum-derived binders in pavement engineering. This interest stems from the recognition that bitumen is produced from non-renewable resources and from the opportunity to utilize agricultural and agro-industrial by-products [16]. Hence, biomodifiers have emerged as environmentally friendly and sustainable alternatives to traditional modifiers. In general, biomodifiers can be classified into two main categories: bio-oils and bio-powders. Among them, bio-powders are of particular interest because they are often obtained as by-products of the agri-food industry. They have attracted considerable attention due to their compatibility with bitumen and their ability to improve binder performance [2]. All starches consist of two structurally distinct components known as amylose and amylopectin. Amylose molecules are primarily composed of glucose units linked by α-D-(1→4) glycosidic bonds and possess molecular weights ranging from 1.3 × 10^5^ to 5 × 10^5^ g/mol. In contrast, amylopectin is a highly branched polysaccharide composed of glucose units connected through both α-D-(1→4) and α-D-(1→6) glycosidic linkages. The molecular weight of amylopectin typically ranges from approximately 10^7^ to 10^9^ g/mol [17,18]. In most starches, amylopectin accounts for approximately 72–82% of the total composition, whereas amylose constitutes about 18–33% [19]. The relative content of these two major components depends on the botanical source, cultivar, and cultivation conditions. From a nutritional perspective, starch is considered a relatively poor source of nutrients because it consists predominantly of carbohydrates and contains only minor amounts of minerals, proteins, and dietary fiber originating from impurities. Nevertheless, starch exhibits excellent thickening, binding, and stabilizing properties, making it a widely used additive in various food products, including bakery products and ready-to-eat foods, where it serves as a binder, thickener, preservative, and texturizing agent [20]. Although native starch possesses numerous functional properties, it also exhibits several limitations, including sensitivity to pH, poor paste clarity, low thermal stability, a high tendency toward retrogradation, and limited solubility [21,22]. Globally, starch is produced primarily from corn and is one of the most abundant biopolymers found in nature. It occurs in the form of microscopic white granules that are insoluble in alcohol, ether, and cold water, while being sensitive to heat and shear forces [23]. Because starch is available as a fine free-flowing powder, it can be incorporated directly into bitumen without the need for any preliminary processing prior to modification. When heated in water, starch gradually absorbs moisture and swells, resulting in an increase in the viscosity of the mixture. Upon further heating, the swollen granules lose their structural integrity, and both amylose and amylopectin become soluble in the hot medium [14]. These properties make starch an attractive material for various industrial applications, including its use as a bio-based modifier for asphalt binders. Although starch is widely recognized as a thickening agent in aqueous systems, its role in bitumen differs fundamentally because no dissolution or gelatinization occurs within the hydrophobic binder matrix. Instead, starch is present as a dispersed bio-based particulate modifier capable of interacting with the polar fractions of bitumen, including resins and asphaltenes [14,24,25]. Such interactions may influence the colloidal organization, restrict molecular mobility, modify oxygen diffusion pathways, and consequently affect the kinetics of thermo-oxidative aging. Therefore, the selection of corn starch in the present study was motivated by its potential to modify the aging behavior of bitumen rather than by its conventional thickening mechanism observed in water-based systems. In response to the growing demand for effective and economically viable additives capable of mitigating the aging of asphalt binders, the primary objective of this study was to determine the kinetic parameters governing the aging process of corn starch-modified bitumen. Although previous studies have demonstrated that starch can improve selected rheological properties of bitumen and asphalt mixtures [14,25], its influence on the kinetics of thermo-oxidative aging has not yet been comprehensively investigated. In particular, there is a lack of studies integrating chemical (FTIR), rheological (dynamic viscosity and MSCR) and kinetic analyses to quantify how starch content affects aging mechanisms under different thermal conditions. Furthermore, the temperature dependence of these processes and their apparent activation energies have not previously been systematically evaluated using the Arrhenius approach. Therefore, the present study aims to fill this research gap by providing a comprehensive kinetic description of the chemical and rheological aging of corn starch-modified bitumen. The working hypothesis of this study is that the incorporation of corn starch modifies the kinetics of aging process by altering the physicochemical interactions within the bitumen matrix. It is assumed that dispersed starch particles influence the colloidal organization of the binder, affecting molecular mobility and oxygen diffusion, which changes the rates of chemical and rheological aging processes. Furthermore, it is hypothesized that these effects are temperature-dependent and therefore may lead to different aging kinetics under moderate (100 °C) and severe (140 °C) aging conditions. It is also expected that the incorporation of corn starch modifies the apparent activation energies of the investigated aging reactions, thereby changing their temperature sensitivity. Finally, it is hypothesized that, within the investigated concentration range, one starch dosage may provide a more favorable balance between aging behavior and technological performance than the other formulations. To verify these hypotheses, the present study quantitatively evaluates the influence of corn starch concentration and aging temperature on the chemical and rheological aging kinetics of paving-grade bitumen using viscosity measurements, FTIR spectroscopy, MSCR testing and Arrhenius analysis. The investigation involved the determination of the rate constants for changes in dynamic viscosity (k_η_), carbon oxidation reactions (k_IC=O_), sulfur oxidation reactions (k_IS=O_), and the aromatization process. In addition, the rate constants describing changes in rheological properties were determined based on the Jnr_3.2_ (k_Jnr3.2_) and R_3.2_ (k_R3.2_) parameters obtained from Multiple Stress Creep Recovery (MSCR) tests.

## 2. Materials and Methods

### 2.1. Materials

**Figure 1:**
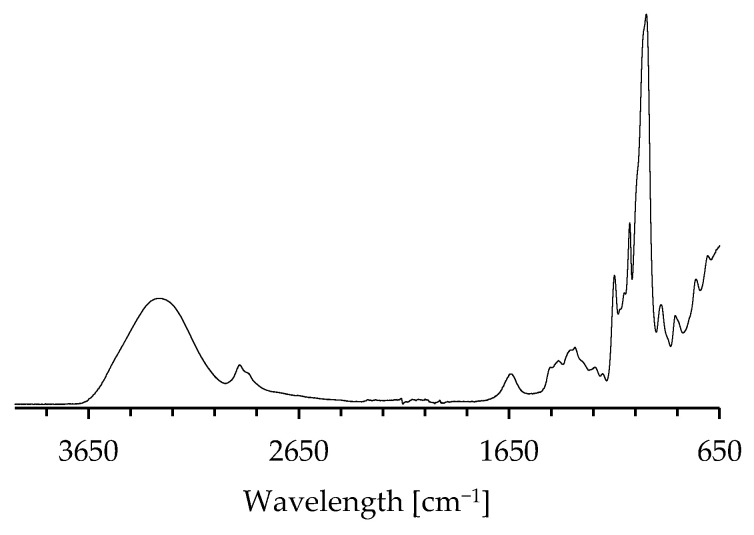
FTIR spectra of corn starch applied in bitumen modification.

**Figure 2:**
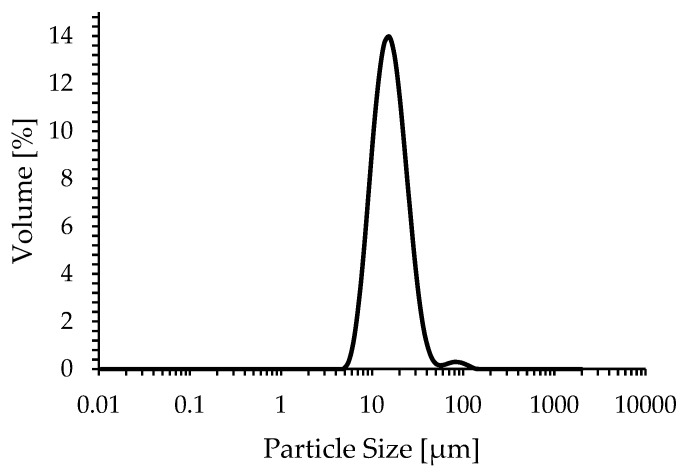
Particle size distribution of the corn starch used as a bitumen modifier, determined by laser diffraction analysis.

The base material selected for modification was 50/70 road asphalt produced by Orlen Asphalt S.A. The reference 50/70 bitumen was characterized in terms of penetration, softening point, and Brookfield viscosity (Table 1). Corn starch (CAS:9005-25-8, EC: 232-679-6) used in this study was purchased from Biomus (Bialystok, Poland). The material was supplied as a high purity, white powder with water content of 13% and was used as received without any further purification. The chemical structure of the corn starch used in this study was characterized by Fourier Transform Infrared (FTIR) spectroscopy (Thermo Fisher Scientific, Madison, WI, USA) (Figure 1). The spectrum exhibited a broad absorption band in the range of 3600–3000 cm^−1^, corresponding to the stretching vibrations of hydroxyl (–OH) groups involved in extensive intra- and intermolecular hydrogen bonding within the amylose and amylopectin structure. A weak absorption band at approximately 2930 cm^−1^ was assigned to the stretching vibrations of aliphatic C–H bonds. The band observed near 1640 cm^−1^ was attributed to the bending vibration of adsorbed water molecules, which is characteristic of starch materials due to their hygroscopic nature. The fingerprint region (1200–900 cm^−1^) was dominated by several intense absorption bands associated with C–O, C–C and C–O–C stretching vibrations of the glucopyranose rings and α-(1→4)/α-(1→6) glycosidic linkages characteristic of starch polysaccharides. The strong absorption bands located at approximately 1150, 1080, 1020 and 995 cm^−1^ confirmed the typical molecular structure of corn starch reported in the literature. The FTIR spectrum therefore verified the presence of abundant hydroxyl-containing polysaccharide chains, which are expected to influence the physicochemical interactions between starch and the bitumen matrix during thermo-oxidative aging. The particle size distribution of the corn starch used in this study is presented in Figure 2. The material exhibited a narrow, unimodal particle size distribution, indicating a relatively homogeneous particle population. The dominant fraction was centered at approximately 13–15 μm, where the maximum volume contribution reached nearly 14%. Most particles were distributed within the range of approximately 8–30 μm, confirming a relatively uniform particle size. A very small secondary population of larger particles was observed at approximately 80–100 μm, although its volume contribution was negligible (<0.5%). This fraction is most likely associated with starch particle agglomerates rather than individual starch granules. The absence of a significant coarse fraction demonstrates that the investigated corn starch possessed a relatively uniform granulometric composition, which is advantageous for achieving homogeneous dispersion within the bitumen matrix during the modification process. Such a particle size distribution is expected to promote good contact between starch particles and the binder, thereby facilitating physicochemical interactions while minimizing the risk of local heterogeneities.

### 2.2. Bitumen Modification

Road bitumen modification process was carried out by adding 4%, 6%, and 8% by weight of corn starch to a 50/70 bitumen matrix. The selected starch contents were based on previously published studies investigating starch as a bio-based modifier for asphalt binders [29,30]. The modification process involved liquefying the base bitumen at 160 °C, followed by the addition of the appropriate amount of biopolymer and homogenization of the mixture at 160 °C for 60 min using a high-speed IKA T-50 mixer (IKA, Staufen im Breisgau, Germany) operating at 4000 rpm. The labels of the obtained bitumen samples are summarized in Table 2.

### 2.3. Aging Simulations

The 50/70 paving-grade bitumen and bitumen modified with corn starch were subjected to thermo-oxidative aging under laboratory conditions. For this purpose, 50 g of bitumen binder was weighed into metal vessels with a diameter of 140 mm. For each type of binder, two independent test series were prepared corresponding to aging temperatures of 100 °C and 140 °C; samples were removed from the heating chamber after 0 h, 24 h, 48 h, 72 h, 96 h, and 120 h of aging. Unlike standardized aging procedures such as RTFOT and PAV, which are primarily intended to simulate technological and long-term field aging, the present study employed controlled isothermal aging to enable a quantitative kinetic analysis. The temperatures of 100 °C and 140 °C were selected to represent two different aging regimes and to allow evaluation of temperature sensitivity using the Arrhenius approach. The aging duration of up to 120 h provided sufficient temporal resolution for determining reaction rate constants and apparent activation energies from time-dependent chemical and rheological measurements. As a result, independent aging series were obtained for each bitumen type and aging temperature, including reference samples (Ref) and bitumen modified with 4% *w*/*w* (SC4), 6% *w*/*w* (SC6), and 8% *w*/*w* (SC8) corn starch. After each aging stage, the obtained samples were subjected to viscosity measurements, as well as analyses of chemical structure (Section 2.5) and rheological properties (Section 2.6).

### 2.4. Viscosity-Based Aging Evaluation

Dynamic viscosity was determined for all samples before and after aging using a Brookfield rotational viscometer Brookfield DV3T (Brookfield, Middleboro, MA, USA) equipped with a Thermosel heating system and SC4-21 spindle. Measurements were carried out at 160 °C in accordance with ASTM D4402. The spindle rotational speed was set at 100 rpm. All dynamic viscosity measurements were performed in triplicate, and the reported values represent the mean of three independent measurements. The measurement variability was evaluated using the standard deviation.

### 2.5. FT-IR Analysis

The chemical and structural composition of both unaged and aged bitumen samples was investigated using Fourier Transform Infrared Spectroscopy (FTIR). The spectra were recorded in triplicate using a Nicolet 380 spectrometer (Thermo Fisher Scientific, Madison, WI, USA) with an ATR technique (Attenuated Total Reflectance) in the wavenumber range of 4000–650 cm^−1^. Measurements were performed at a resolution of 6 cm^−1^ with 64 scans for each sample. The FTIR spectra were processed using OMNIC software ver. 6.x (Thermo Fisher Scientific, Madison, WI, USA) without baseline correction. The spectra were subsequently used for the determination of characteristic oxidation indices. The carbonyl (I_C=O_), sulfoxide (I_S=O_) and aromaticity (I_C=C_) indices were calculated as the ratio of the integrated area of the characteristic absorption band to the total integrated spectral area (ΣA) according to Equations (1)–(3). The integrated peak areas were determined using the following spectral ranges: 1750–1650 cm^−1^ for carbonyl groups, 1080–980 cm^−1^ for sulfoxide groups and 1650–1550 cm^−1^ for aromatic C=C stretching vibrations. The total spectral area (ΣA) was calculated as the sum of the integrated peak areas within the following spectral regions: 3600–3100, 3100–2700, 1750–1650, 1650–1550, 1520–1400, 1400–1320, 1320–1270, 1180–1140, 1140–1120, 1080–1045, 1080–980 and 780–740 cm^−1^.

(1) $$I_{C=O}=\frac{A_{1700}}{\Sigma A}$$

(2) $$I_{S=O}=\frac{A_{1030}}{\Sigma A}$$

(3) $$I_{C=C}=\frac{A_{1600}}{\Sigma A}$$

### 2.6. Rheological Analysis

Rheological properties were evaluated for all samples using an Anton Paar MCR302e Dynamic Shear Rheometer (Graz, Austria). Multiple Stress Creep Recovery (MSCR) analysis was carried out in accordance with AASHTO TP 350 [31] at 64 °C using parallel DSR plates with a diameter of 25 mm and 1 mm gap. The samples were tested in triplicate in creep and recovery states under the application of stress load of 3.2 kPa. The obtained results were used to determine the non-recoverable creep compliance (J_nr_) and percent recovery (R) using Equations (4) and (5). The analysis was limited to the 3.2 kPa stress level because the primary objective of this study was the comparative assessment of permanent deformation resistance using the Jnr_3.2_ parameter [32,33,34], which is recognized as the principal MSCR indicator for rutting performance and forms the basis for binder classification. The lower stress level (0.1 kPa) was not included, as the stress sensitivity parameter (Jnr, diff) was beyond the scope of the present investigation.

(4) $$J_{nr}=\frac{\varepsilon_{nr}}{\sigma }$$

(5) $$R=\frac{\varepsilon_{\max}-\varepsilon_{nr}}{\varepsilon_{\max}}\times 100\%$$

## 3. Results and Discussion

### 3.1. Viscosity-Based Analysis

**Figure 3:**
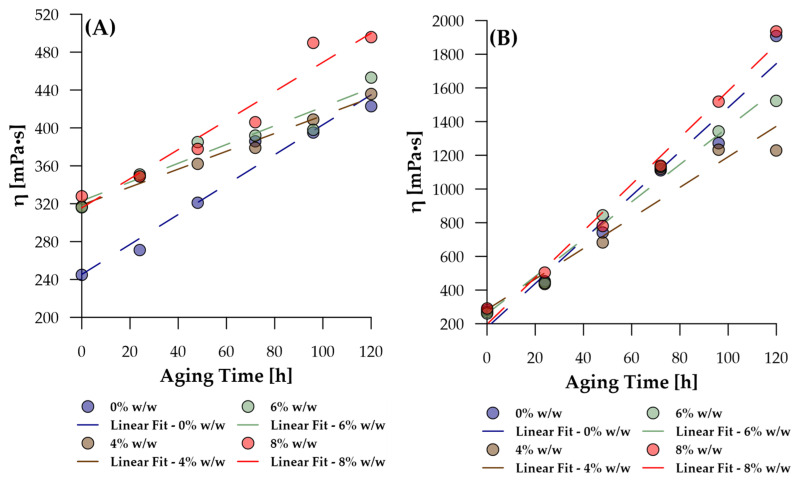
Changes in the dynamic viscosity of the investigated binders during aging at 100 °C (**A**) and 140 °C (**B**).

An important consequence of bitumen aging is the progressive increase in bitumen viscosity resulting from oxidation reactions, volatilization of light fractions and structural transformations occurring within the colloidal system of the bitumen binder. These processes promote the formation of larger and more polar molecular structures, leading to enhanced intermolecular interactions and binder hardening [35,36]. To quantitatively compare the aging behavior of the investigated binders, empirical (apparent) kinetic models were adopted to describe the temporal evolution of the measured chemical and rheological parameters. The selection of the kinetic model was based on the experimental trends observed during aging. Parameters exhibiting approximately linear changes with aging time were described using apparent zero-order kinetics, whereas the evolution of Jnr_3_._2_ was represented by a first-order logarithmic model. It should be emphasized that these models are intended as empirical descriptions of the experimental data for comparative purposes rather than mechanistic models describing the elementary oxidation reactions occurring in bitumen. Figure 3A,B presents the evolution of dynamic viscosity during bitumen aging at T = 100 °C and T = 140 °C. The experimental data were successfully described using a zero-order kinetic model (Equation (6)), as confirmed by the high coefficients of determination (R^2^ = 0.925–0.985). Therefore, the slope of the regression line was adopted as the apparent rate constant of viscosity increase (k_η_), allowing quantitative evaluation of the aging kinetics.

(6) $$∆\eta =\eta_{t}-\eta_{0}=k_{\eta }\cdot t$$

At 100 °C (Figure 3A), all investigated bitumen binders exhibited a nearly linear increase in viscosity with aging time. The dynamic viscosity changes in reference bitumen was described by the equation η = 1.5798∙t + 245.38 (R^2^ = 0.9610), yielding a viscosity growth rate constant k_η_ of 1.5798 mPa·s h^−1^. A considerably lower rate of viscosity increase was observed for SC4 and SC6, whose aging behavior was described by the equations η = 0.9429∙t + 318.76 (R^2^ = 0.9846) and η = 0.9917∙t + 323.00 (R^2^ = 0.9246), corresponding to k_η_ values of 0.9429 and 0.9917 mPa·s h^−1^, respectively. These values show approximately 40% lower aging rates compared with the reference bitumen. In contrast, the SC8 binder exhibited a viscosity increase described by η = 1.5369∙t + 315.62 (R^2^ = 0.9429), resulting in a k_η_ value of 1.5369 mPa·s h^−1^, very close to k_η_ value determined for the neat, unmodified bitumen. At an aging temperature of 100 °C, the kinetics of dynamic viscosity growth were strongly dependent on the corn starch content in the binder. Based on the determined rate constants, the susceptibility of the binders to viscosity increase decreased in the following order: Ref ≈ SC8 > SC6 > SC4. This suggests that the reference binder and the SC8 binder exhibited the highest rates of viscosity growth, reflecting the most intensive aging processes leading to binder stiffening. In contrast, the SC6 and particularly the SC4 binders showed a noticeable reduction in the rate of viscosity increase, indicating a slowdown of the processes responsible for rheological aging. The obtained results suggest that the addition of corn starch can effectively reduce the rate of dynamic viscosity changes occurring during aging at lower temperatures. The most pronounced retardation of viscosity growth was observed for the SC4 binder, for which the lowest value of kη was determined. At the same time, increasing the starch content resulted in a gradual increase in the rate of viscosity changes, reaching values for the SC8 binder that were comparable to those of the unmodified binder. These trend suggests the existence of an optimum starch content in the binder. At low concentrations, starch may stabilize the colloidal structure of asphalt and retard viscosity growth during aging. However, further increases in starch content may promote particle aggregation and the formation of a more structured binder network, reducing the effectiveness of the modifier and resulting in viscosity growth rates approaching those of the unmodified binder [37,38].

A substantial acceleration of the bitumen aging process was observed when the temperature increased to 140 °C (Figure 3B). The changes in dynamic viscosity of reference bitumen exhibited a kinetic equation of η = 13.06∙t + 177.43 (R^2^ = 0.9616), corresponding to a k_η_ value of 13.06 mPa·s h^−1^. Lower values were determined for SC4 and SC6 bitumen, described by the equations η = 9.069∙t + 284.19 (R^2^ = 0.9261) and η = 11.08∙t + 259.38 (R^2^ = 0.9842), yielding k_η_ values of 9.069 mPa·s h^−1^ and 11.08 mPa·s h^−1^, respectively. The highest viscosity growth rate was observed for SC8, whose aging kinetics were represented by the equation η = 13.852∙t + 196.86 (R^2^ = 0.9872). The resulting k_η_ value of 13.852 mPa·s h^−1^ exceeded even that of the reference bitumen, indicating the highest susceptibility to hardening among all analyzed materials. The conducted studies demonstrated that, at an aging temperature of 140 °C, the influence of corn starch content on the kinetics of dynamic viscosity growth was even more pronounced than at 100 °C. Based on the determined rate constants, the susceptibility of the investigated binders to rheological aging increased in the following order: SC8 > Ref > SC6 > SC4. This demonstrates that the SC8 binder exhibited the highest rate of dynamic viscosity increase, suggesting the most intensive progression of processes leading to the formation of high-molecular-weight structures and an increased degree of networking within the colloidal system [39]. The reference binder exhibited slightly lower susceptibility to aging than the SC8 binder, whereas the SC6 and particularly the SC4 binders showed significantly lower rates of viscosity change during exposure to elevated temperature. These findings suggest that the protective effect of corn starch against degradation at 140 °C is strongly dependent on its concentration in the binder. The most beneficial effect with respect to viscosity growth was observed for the SC4 binder, which exhibited the lowest rate constant of viscosity increase, indicating the greatest resistance to the processes responsible for binder hardening. A protective effect was also observed for the SC6 binder; however, its effectiveness was lower than that of SC4. In contrast, a further increase in starch content to the level corresponding to the SC8 binder resulted in a substantial acceleration of viscosity changes, exceeding even the values obtained for the unmodified binder. This behavior may indicate that excessive amounts of starch promote the formation of high-molecular-weight aging products or intensify the aggregation of asphalt constituents, leading to a more rapid increase in viscosity during aging at 140 °C. Furthermore, starch is a material whose rheological and structural properties are highly temperature-dependent. Under elevated-temperature conditions, processes such as swelling, partial degradation, and structural reorganization of starch may occur, thereby affecting its interactions with the bitumen matrix. Thus, the modification effect is not necessarily proportional to the amount of additive introduced, and once the optimum concentration is exceeded, the beneficial influence of starch may be reduced. The obtained results suggest that the starch content corresponding to the SC8 binder may have exceeded the effective range of modifier performance, resulting in an increased susceptibility of the binder to viscosity growth during aging [37,40,41]. The observed reduction in viscosity growth for the SC4 and SC6 binders may also be explained by changes in the colloidal organization of the bitumen system. Corn starch particles may preferentially interact with the polar maltene fractions, reducing molecular mobility and slowing down the aggregation of oxidized species into larger asphaltene-like structures. Consequently, the formation of a continuous network of high-molecular-weight oxidation products is delayed, resulting in a slower increase in binder viscosity [42,43]. At excessive starch contents, however, particle agglomeration and reduced compatibility with the bitumen matrix may weaken this stabilizing effect, explaining the higher viscosity growth observed for the SC8 binder. The standard deviations of the viscosity measurements (Table 3) indicate good repeatability of the experimental results. The determined rate constants reveal that aging temperature was the primary factor controlling the kinetics of viscosity growth.

The determined rate constants reveal that aging temperature was the primary factor controlling the kinetics of viscosity growth. As shown in Figure 3, increasing the aging temperature from 100 °C to 140 °C resulted in an approximately 8–11-fold increase in the rate constant for all investigated binders. The relatively small differences in the k140/k100 ratio suggest that the temperature sensitivity of the rheological aging process remained similar regardless of the corn starch content. Increasing the aging temperature from 100 °C to 140 °C resulted in an approximately 9-fold increase in k_η_ for the reference binder, a 9.6-fold increase for SC4, an 11.2-fold increase for SC6 and a 9.0-fold increase for SC8. These observations confirm the strong temperature dependence of the aging process and confirm that the effectiveness of the applied modifiers differs under severe thermal conditions. Among the investigated materials, SC4 exhibited the highest resistance to aging-induced viscosity growth at both temperatures, whereas SC8 showed aging behavior comparable to or even more pronounced than that of the unmodified binder.

The aging resistance of starch-modified bitumen is governed by the behavior of the composite binder rather than by the intrinsic thermal stability of starch alone. Although starch itself is susceptible to thermal degradation, its incorporation modifies the colloidal organization of the binder and influences the kinetics of oxidation reactions. Consequently, the overall aging behavior results from the interaction between starch and bitumen constituents, leading to either acceleration or inhibition of specific aging processes depending on starch concentration and aging temperature.

### 3.2. Oxidation of Carbon Atoms in Road Bitumen Hydrocarbons

**Figure 4:**
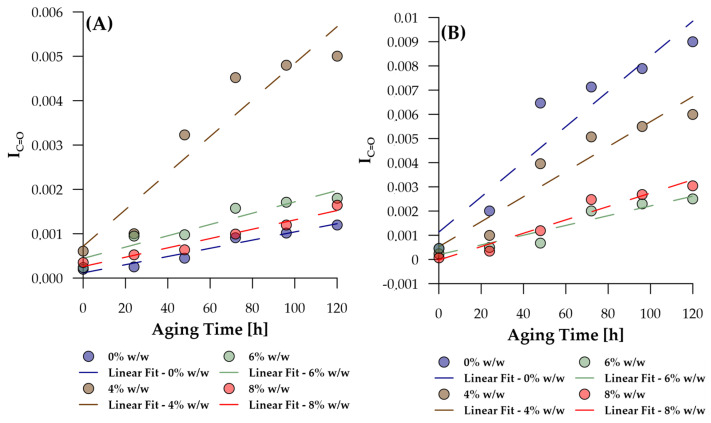
Changes in the carbonyl index (I_C=O_) of the investigated binders during aging at 100 °C (**A**) and 140 °C (**B**).

The oxidative aging of bitumen is directly associated with reactions between atmospheric O_2_ and carbon atoms present in the hydrocarbon structures of the binder. Under elevated temperatures, dehydrogenation of bitumen molecules occurs, leading to the formation of carbon-centered radicals that initiate further oxidation reactions. As a result, polar carbonyl groups (C=O) are formed, increasing the polarity and association degree of bitumen constituents. The oxidation leads to an increase in binder viscosity and stiffness, accompanied by a deterioration of its performance-related properties [39,44]. An increase in the carbonyl index is widely recognized as a measure of the extent of oxidative degradation of bitumen, as it reflects the degree of transformation of asphalt constituents into more polar oxygen-containing compounds. Since carbonyl compounds constitute the primary oxidation products generated during bitumen aging, changes in the I_C=O_ value can be directly associated with the advancement of thermo-oxidative degradation processes. As presented in Figure 4, the increase in carbonyl index over aging time followed a linear trend for all investigated bitumen binders, indicating that the formation of carboxyl groups can be described using a zero-order kinetic model. The high values of the determination coefficients (R^2^ = 0.891–0.962) confirmed the applicability of this model and allow the slope of the regression line to be interpreted as the apparent carbonyl formation rate constant$\;k_{I_{C=O}}$ as defined by Equation (7).

(7) $$∆I_{C=O}==I_{C=O(t)}-I_{C=O(0)}=k_{I_{C=O}}\cdot t$$

The oxidation kinetics during aging at T = 100 °C revealed that the SC4 binder was characterized by the fastest formation of carbonyl groups, indicating the highest rate of carbon atom oxidation (Figure 4A). The experimental data were described by the equation I_C=O_ = 4 × 10^−5^∙t + 0.0005 with R^2^ = 0.9061, corresponding to a $k_{I_{C=O}}$ of 4 × 10^−5^ 1/h. This value was approximately five times higher than that determined for the unmodified bitumen, whose oxidation behavior was represented by I_C=O_ = 9 × 10^−6^·t + 0.0001 (R^2^ = 0.9534). For SC6 and SC8 bitumen binders an intermediate carbon atoms oxidation rate $k_{I_{C=O}}$ = 1 × 10^−5^ 1/h were determined. Based on the determined $k_{I_{C=O}}$ values, the rate of carbon atom oxidation during aging at T = 100 °C followed the order Ref < SC8 ≈ SC6 < SC4, demonstrating a progressive increase in oxidation susceptibility with increase in corn starch amount, reaching its maximum for the SC4 sample. The results suggest that at lower aging temperature the incorporation of starch initially promotes the formation of oxidation products, although this effect diminishes as the starch content increases. This behavior may be related to the presence of hydroxyl-rich starch particles, which can alter the interactions between polar asphalt constituents and influence oxidation reactions. The reduced effect observed at higher starch contents may show the existence of an optimum modifier concentration, beyond which particle aggregation or reduced compatibility with the binder limits the influence of starch on the aging process [45,46].

A noticeably different aging behavior of neat and corn starch-modified road bitumen binders was observed during their aging at T = 140 °C (Figure 4B). The reference bitumen displayed the highest susceptibility to oxidative transformation, yielding a kinetic equation of I_C=O_ = 7 × 10^−5^·t + 0.0011 (R^2^ = 0.8987). In contrast, all starch-modified binders exhibited lower carbonyl formation rates. The C-atoms oxidation of SC4 binder was characterized by $k_{I_{C=O}}$ of 4 × 10^−5^ 1/h, whereas the values determined for SC8 and SC6 were 3 × 10^−5^ 1/h and 10^−5^ 1/h, respectively. The obtained results showed that, at bitumen aging temperature of 140 °C, the rate of carbon atom oxidation increased in the following order: SC6 < SC8 < SC4 < Ref, indicating that the SC6 binder exhibited the highest resistance to carbonyl group formation, whereas the reference binder was the most susceptible to thermo-oxidative degradation. Particularly noteworthy is the behavior of SC6, for which the lowest rate constant was determined despite the relatively high oxidation rate observed during aging at T = 100 °C. This finding demonstrates that the protective effect of corn starch becomes increasingly pronounced at elevated temperatures and may be associated with starch-induced modifications of the binder microstructure. Such structural changes can influence the mobility and interactions of asphalt constituents, thereby affecting the course of aging process [45]. From a physicochemical perspective, carbonyl formation is governed not only by the intrinsic oxidation susceptibility of bitumen molecules but also by oxygen transport through the binder [47,48]. Starch-induced changes in the microstructure may reduce oxygen diffusion pathways and increase the tortuosity of the oxidation front, thereby slowing the formation of oxygen-containing functional groups. Similar effects have been reported for other bio-based modifiers, where modification of the colloidal structure rather than direct chemical reaction with bitumen was identified as the primary mechanism responsible for improved aging resistance [49,50].

The comparison of kinetic parameters obtained at both temperatures for the analyzed aging process reveals that the influence of corn starch on carbonyl generation is strongly temperature-dependent. At T = 100 °C, moderate corn starch contents enhanced the formation of oxidation products, whereas at T = 140 °C all modified bitumen binders exhibited improved resistance to oxidation relative to the reference road bitumen. The greatest carbon oxidation-inhibiting effect was observed for SC6, which combined a low carbonyl formation rate with the highest reduction in temperature sensitivity. These observations suggest that starch not only modifies the physical structure of the bitumen binder but may also participate in mechanisms that modify the oxidation pathway of bitumen components. As a result, the optimum starch concentration appears to provide a balance between initial oxidative activity and long-term resistance to oxidation process, although this optimum depends on the aging indicator and aging temperature considered. The repeatability of the carbonyl index measurements was evaluated based on triplicate FTIR analyses, and the corresponding standard deviations are presented in Table 4.

The temperature sensitivity of carbonyl group formation within investigated bitumen binders during aging was further evaluated using the Arrhenius approach. The activation energies Ea (Table 5) were calculated from the dependence of the logarithm of the reaction rate constant on the reciprocal absolute aging temperature. Positive activation energy values were obtained for all investigated binders, confirming that the formation of carbonyl-containing oxidation products is a thermally activated process and that increasing the aging temperature accelerates the oxidation reactions occurring within the binder structure [51]. The calculated activation energies were equal to 67.6 kJ/mol, 17.1 kJ/mol, 22.1 kJ/mol and 35.4 kJ/mol for the Ref, SC4, SC6 and SC8 bitumen binders, respectively. Consequently, the activation energy increased in the order SC4 < SC6 < SC8 < Ref. The highest activation energy determined for the reference bitumen indicates that the rate of carbonyl formation within its structure is strongly dependent on temperature. In contrast, the starch-modified bitumen binders exhibited noticeably lower activation energies, suggesting that the incorporation of starch reduced the temperature sensitivity of the oxidation process. Among the modified binders, the lowest activation energy was observed for SC4, indicating that the oxidation reactions responsible for carbonyl generation required the smallest energy input to proceed. At the same time, SC4 was characterized by the highest carbonyl formation rate constants at both aging temperatures, demonstrating that activation energy alone should not be considered a direct measure of aging resistance. Instead, the experimental evidence suggests that starch modification affects both the intrinsic kinetics of oxidation and the temperature dependence of the process. The intermediate activation energies determined for SC6 and SC8 show that increasing starch content alters the thermo-oxidative behavior of the binder, resulting in a gradual increase in the energy barrier associated with carbonyl formation. This confirms that starch influences not only the rate of oxidation reactions but also their sensitivity to temperature, thereby modifying the overall aging mechanism of the investigated binders. These variations in activation energy indicate that the presence of starch modifies the aging kinetics and may alter the dominant pathways in the investigated bitumen binders [52]. It should be noted that the Arrhenius analysis performed in this study was based on only two aging temperatures (100 °C and 140 °C). Consequently, the calculated activation energies should be interpreted as comparative estimates of the relative temperature sensitivity of the investigated binders rather than as precisely determined intrinsic activation energies. Although this approach is sufficient for comparing the influence of corn starch on aging kinetics, future studies involving a wider range of aging temperatures would allow a more rigorous validation of the Arrhenius relationship and a more accurate determination of the activation energy. It should be emphasized that the apparent activation energy reflects the temperature sensitivity of the carbonyl formation process rather than the aging resistance of the binder itself. Therefore, the activation energy should be interpreted together with the corresponding apparent rate constants, as both parameters jointly describe the thermo-oxidative behavior of the investigated binders. Consequently, a higher activation energy does not necessarily indicate superior aging resistance but rather a stronger dependence of the carbonyl formation rate on temperature.

Although the SC4 binder exhibited the highest apparent carbonyl formation rate at 100 °C, this observation should not be interpreted as evidence of the poorest overall aging resistance. Carbonyl formation represents only one of several parallel thermo-oxidative processes occurring in bitumen. Moreover, at elevated temperatures, oxidation may be accompanied by the volatilization of low-molecular-weight oxygen-containing compounds, meaning that the measured carbonyl index reflects only the oxidation products retained within the binder. Consequently, the overall aging behavior should be evaluated using a combination of chemical, rheological and kinetic parameters rather than a single FTIR indicator. Based on this comprehensive assessment, the binder containing 4 wt.% corn starch provided the most favorable overall aging performance.

### 3.3. Oxidation of Sulfur-Containing Compounds in Road Bitumen

**Figure 5:**
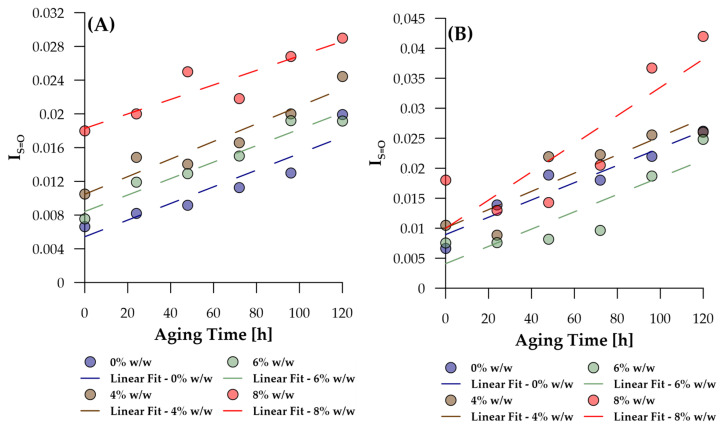
Changes in the sulphoxide index (I_S=O_) of the investigated binders during aging at 100 °C (**A**) and 140 °C (**B**).

The oxidation of sulfur-containing compounds naturally present in road bitumen results in the formation of sulfoxide (S=O) functional groups, which are also considered as an indicator of oxidative aging. The formation of S=O groups increase the polarity of bitumen components and promotes stronger intermolecular interactions, thereby contributing to binder hardening. Thus, monitoring sulfoxide formation provides valuable information on the progress of oxidative degradation occurring during bitumen aging [39,44,53]. Figure 5A,B illustrates the evolution of the sulfoxide index during bitumen aging. A nearly linear increase in the sulfoxide index was observed for all investigated bitumen binders, indicating that the oxidation of sulfur-containing compounds can be adequately described by a zero-order kinetic model (Equation (8)). The high coefficients of determination (R^2^ = 0.837–0.950) confirm the suitability of this approach and allow the slope of the regression line to be interpreted as the apparent sulfoxide formation rate constant ($k_{I_{S=O}}$).

(8) $$∆I_{S=O}==I_{S=O(t)}-I_{S=O(0)}=k_{I_{S=O}}\cdot t$$

The kinetic analysis of bitumen aging performed at T = 100 °C (Figure 5A, Table 6) revealed comparable rates of sulfoxide index evolution among all investigated binders. The reference bitumen was characterized by the kinetic equation of S-atom oxidation I_S=O_ = 9.87 × 10^−5^∙t + 0.0114 (R^2^ = 0.9459), corresponding to a sulfoxide formation rate constant of $k_{I_{S=O}}$ = 9.87 × 10^−5^ 1/h. Whereas, SC4 and SC6 are described by the equations I_S=O_ = 1.04 × 10^−4^∙t + 0.0105 (R^2^ = 0.9177) and I_S=O_ = 9.77 × 10^−5^∙t + 0.0084 (R^2^ = 0.9406), respectively. A slightly lower value was observed for SC8, whose oxidation behavior was represented by I_S=O_ = 8.60 × 10^−5^∙t + 0.0183 (R^2^ = 0.8411). The observed behavior indicates that, under moderate aging conditions, corn starch addition had only a limited influence on the oxidation kinetics of sulfur-containing bitumen components. The similar $k_{I_{S=O}}$ values obtained for major bitumen binders suggest that the initial stages of sulfur oxidation are governed primarily by the intrinsic chemical composition of the bitumen rather than by the modifier content. A substantially different behaviour of analyzed bitumen samples was observed after aging at T = 140 °C, where the influence of corn starch concentration became more pronounced. The reference bitumen binder exhibited the lowest sulfoxide formation rate among all investigated materials, with a kinetic equation of I_S=O_ = 1.17 × 10^−4^∙t + 0.0054 (R^2^ = 0.8686) and a corresponding rate constant of $k_{I_{S=O}}$ = 1.17 × 10^−4^ 1/h. In contrast, the starch-modified binders showed markedly higher oxidation rates. The SC4 and SC6 binders were described by the equations I_S=O_ = 1.72 × 10^−4^∙t + 0.0083 (R^2^ = 0.8457) and I_S=O_ = 1.53 × 10^−4^∙t + 0.0033 (R^2^ = 0.8365), respectively, corresponding to $k_{I_{S=O}}$ = 1.72 × 10^−4^ 1/h and $k_{I_{S=O}}$ = 1.53 × 10^−4^ 1/h, respectively. The highest sulfoxide formation rate was observed for SC8, whose aging kinetics were represented by the equation I_S=O_ = 2.69 × 10^−4^x + 0.0070 (R^2^ = 0.8630). The resulting rate constant of $k_{I_{S=O}}$ = 2.69 × 10^−4^ 1/h was approximately three times greater than that determined for the reference bitumen, indicating a significantly enhanced susceptibility to sulfur oxidation at an elevated temperature.

The comparison of kinetic parameters obtained at both aging temperatures demonstrates that the influence of corn starch on sulfoxide formation is strongly temperature-dependent. While rates of sulfur oxidation during aging at T = 100 °C remained relatively similar regardless of corn starch content, increasing the aging temperature led to a progressive increase in $k_{I_{S=O}}$ with increasing starch dosage. The most pronounced effect was observed for SC8, which exhibited the highest sulfoxide formation rate and the largest increase in temperature sensitivity. This behaviour suggests that elevated starch contents may facilitate the formation of oxygenated sulfur compounds during aging, possibly by increasing oxygen accessibility within the binder structure or by promoting reactions involving sulfur-containing oxidation intermediates. Although starch modification may improve selected performance characteristics of bitumen, excessive starch content appears to accelerate the oxidation of sulfur-containing compounds under severe aging conditions. The different behavior of sulfoxide formation compared with carbonyl formation indicates that these oxidation pathways are influenced to different extents by starch modification. Sulfoxide groups originate mainly from the oxidation of sulfur-containing compounds naturally present in bitumen, whereas carbonyl groups are formed through the oxidation of hydrocarbon chains. Consequently, the modifier may affect these two processes differently by altering oxygen accessibility and molecular mobility within the binder matrix [54,55].

The temperature sensitivity of sulfur oxidation reactions was evaluated using the Arrhenius equation based on the rate constants of sulfoxide group formation. The calculated apparent Ea values were equal to 5.33 kJ/mol, 16.03 kJ/mol, 14.42 kJ/mol and 36.53 kJ/mol for corn starch amount of 0% *w*/*w*, 4% *w*/*w*, 6% *w*/*w* and 8% *w*/*w*, respectively. Consequently, the activation energy increased in the order Ref < SC6 < SC4 < SC8. Positive activation energy values obtained for all investigated binders confirm that the formation of sulfoxide groups is a thermally activated process and that increasing the aging temperature promotes the oxidation of sulfur-containing compounds present in the bitumen structure. The lowest activation energy determined for the reference binder confirms that the rate of sulfur oxidation exhibited only a limited dependence on temperature within the investigated range. In contrast, starch-modified binders were characterized by considerably higher activation energies, suggesting that starch incorporation increased the energy barrier associated with the formation of sulfoxide groups. Among the modified binders, the highest activation energy was obtained for SC8, indicating the strongest temperature dependence of sulfur oxidation reactions. This behavior may be attributed to the influence of starch on the microstructural organization of the binder, which can modify oxygen diffusion pathways and alter the accessibility of sulfur-containing species to oxidative attack. The observed increase in activation energy with starch content suggests that the modifier affects not only the rate of sulfur oxidation but also the mechanism governing the formation of oxidation products. Since sulfoxide groups originate exclusively from sulfur-containing compounds naturally present in bitumen, the obtained results provide direct evidence that starch modification alters the aging behavior of the bitumen binder. Furthermore, the significantly higher activation energy determined for SC8 suggests that the oxidation process becomes increasingly temperature-controlled as the starch content increases, which may contribute to improved resistance against sulfur oxidation under moderate service temperatures. Similarly, the apparent activation energy determined for sulfoxide formation (Table 7) should be regarded as a measure of the temperature dependence of this oxidation pathway rather than as an independent indicator of aging resistance. A comprehensive assessment of the thermo-oxidative behavior requires simultaneous consideration of both the activation energy and the experimentally determined sulfoxide formation rate constants. Therefore, the aging performance of the investigated binders cannot be inferred solely from the activation energy values.

### 3.4. Aromatization of Aliphatic Components of Road Bitumen

**Figure 6:**
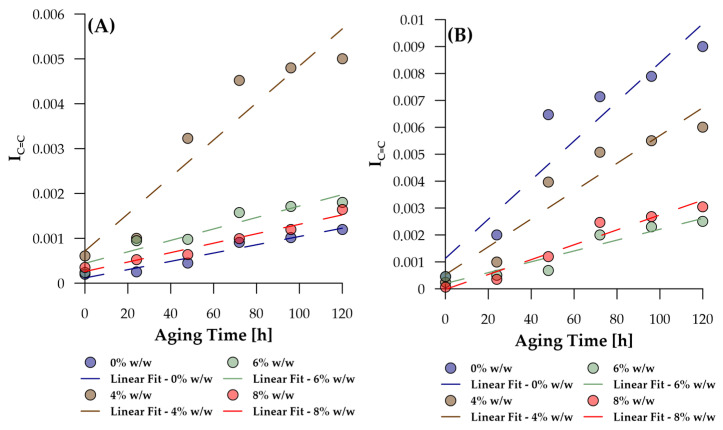
Changes in the aromaticity index (I_C=C_) of the investigated binders during aging at 100 °C (**A**) and 140 °C (**B**).

Apart from oxidation reactions, the constituents of bitumen also undergo aromatization. This process results in the transformation of aliphatic structures into thermodynamically more stable aromatic structures and may be induced by both elevated temperature and ultraviolet (UV) radiation. It is associated with the dehydrogenation of cycloaliphatic rings accompanied by an increase in the number of conjugated π bonds. An increase in the aromatization degree of bitumen components affects its physicochemical properties by enhancing polarity, chemical reactivity and susceptibility to further oxidative transformations. Consequently, aromatization is considered one of the mechanisms responsible for the increase in stiffness, viscosity and overall aging severity of road bitumen [56,57]. The aromaticity index (I_aromaticity_) is an important parameter describing aromatization process occurring in bitumen during its oxidative aging. Changes in aromaticity are closely associated with molecular rearrangement reactions, which alter the balance between aromatic, resins and asphaltenes fraction of bitumen. Hence, monitoring the evolution of I_aromaticity_ provides valuable information regarding the chemical stability of modified binders occurring during thermal aging. As shown in Figure 6, the aromaticity index increased almost linearly with aging time for all investigated binders, and the obtained data were satisfactorily described using a zero-order kinetic model (Equation (9)), with coefficients of determination ranging from R^2^ = 0.875 to R^2^ = 0.956. Therefore, the slope of each regression line was adopted as the apparent rate constant of aromaticity development $k_{I_{aromatization}}$.

(9) $$∆I_{aromaticity}==I_{aromaticity(t)}-I_{aromaticity(0)}=k_{I_{aromatization}}\cdot t$$

As is seen on Figure 6A, the highest rate of aromaticity process during aging at T = 100 °C was observed for the SC8 bitumen. The change in I_aromaticity_ in aging time was described by the equation I_aromaticity_ = 1.00 × 10^−4^∙t + 0.0032 (R^2^ = 0.9313), corresponding to an apparent rate constant of $k_{I_{aromatization}}$ = 1.00 × 10^−4^ 1/h. Slightly lower values of $k_{I_{aromatization}}$ were determined for SC4 and reference bitumen, whose kinetic equations were I_aromaticity_ = 9.68 × 10^−5^∙t + 0.0035 (R^2^ = 0.9672) and I_aromaticity_ = 7.54 × 10^−4^∙t + 0.0047 (R^2^ = 0.8750), respectively. The corresponding rate constants amounted to 9.68 × 10^−5^ 1/h and 7.54 × 10^−4^ 1/h. The SC6 binder exhibited the lowest aromaticity growth rate, described by the equation I_aromaticity_ = 3.00 × 10^−5^∙t + 0.0140 (R^2^ = 0.9556), yielding $k_{I_{aromatization}}$= 3.00 × 10^−5^ 1/h. These results show that the incorporation of corn starch to bitumen matrix significantly accelerated structural transformations associated with the aromatic fraction during aging at T = 100 °C. The susceptibility to aromaticity process of investigated bitumen binders increased in the following order: SC6 < Ref < SC4 < SC8.

The effect of temperature became more considerable during bitumen aging at T = 140 °C, where substantially different kinetic behavior was observed. The SC6 bitumen exhibited the highest aromaticity growth rate, with a kinetic equation of I_aromaticity_ = 1.585 × 10^−4^∙t + 0.0141) (R^2^ = 0.9313), corresponding to $k_{I_{aromatization}}=\;$1.585 × 10^−4^ 1/h. A comparable value was obtained for SC8 which aromaticity change during aging at T = 140 °C was described using I_aromaticity_ = 1.040 × 10^−4^∙t + 0.0055 (R^2^ = 0.9104) equation, indicating similar susceptibility to aging-induced structural rearrangements. Slightly lower kinetics were recorded for SC4, described by I_aromaticity_ = 0.0023∙t + 0.0033 (R^2^ = 0.9405) equation. In contrast, SC6 exhibited markedly different behavior, with the lowest rate constant among all investigated bitumen binders. The determined equation I_aromaticity_ = 0.0007∙t + 0.0048 (R^2^ = 0.9397) yielded a $k_{I_{aromatization}}$ value of only 7.0 × 10^−4^ 1/h, demonstrating a significant reduction in the rate of aromaticity increase compared with the remaining analyzed bitumen binders.

Comparison of the kinetic parameters determined at both aging temperatures reveals that the influence of corn starch on aromaticity change strongly depends on biopolymer concentration. For SC4 and SC6, increasing the aging temperature resulted in a decrease in the rate constant from $k_{I_{aromatization}}$ = 4.6 × 10^−3^ 1/h to $k_{I_{aromatization}}$ = 2.3 × 10^−3^ 1/h and from $k_{I_{aromatization}}$ = 3.8 × 10^−3^ 1/h to $k_{I_{aromatization}}$ = 7.0 × 10^−4^ 1/h, respectively. The most pronounced reduction was therefore observed for SC6, suggesting enhanced resistance to temperature-induced structural transformations of aromatic compounds. Conversely, the reference binder exhibited an increase in aromaticity growth rate from $k_{I_{aromatization}}$ = 1.8 × 10^−3^ 1/h to $k_{I_{aromatization}}$ = 2.5 × 10^−3^ 1/h, indicating greater susceptibility to bitumen degradation. The behavior of SC8 was particularly noteworthy, as its rate constant remained practically unchanged despite the increase in aging temperature. Overall, the results reveal that corn starch modification substantially affects the aromaticity kinetics during bitumen binders aging. Among the investigated binders, SC6 demonstrated the highest resistance to temperature-induced aromatic transformations, whereas SC8 exhibited behavior similar to that of the unmodified binder under severe aging conditions. The standard deviations of the aromaticity index determined from triplicate FTIR measurements are summarized in Table 8, confirming the good repeatability of the experimental results. Aromatization is generally associated with progressive condensation and structural rearrangement of hydrocarbon molecules during aging [58,59]. These transformations increase the degree of molecular ordering and contribute to the formation of larger aromatic structures. The presence of corn starch may indirectly influence these reactions by modifying the spatial organization of bitumen constituents and reducing the probability of intermolecular interactions leading to aromatic condensation. This may explain the differences in aromatization kinetics observed among the investigated binders.

The temperature dependence of the aromatization process was evaluated using the Arrhenius equation. The calculated apparent activation energies (Table 9) were 4.75, 20.80, 48.42 and 1.31 kJ/mol for the Ref, SC4, SC6 and SC8 binders, respectively. The activation energy increased in the order SC8 < Ref < SC4 < SC6. Positive activation energies obtained for all investigated binders confirm that the transformation of aliphatic structures into aromatic species is a thermally activated process. The lowest activation energies determined for the Ref and SC8 binders demonstrate that the aromatization process exhibited only a weak dependence on temperature within the investigated range. In contrast, considerably higher activation energies were obtained for SC4 and particularly for SC6, suggesting a substantially greater sensitivity of the aromatization reactions to temperature. The highest activation energy observed for SC6 demonstrates that elevated temperatures strongly promote the formation of aromatic structures in this binder. These findings confirm that starch modification alters not only the rate of aromatization but also the energy barrier associated with the transformation of aliphatic hydrocarbons into more condensed aromatic structures during thermo-oxidative aging. The calculated activation energies associated with aromatization primarily characterize the sensitivity of structural transformation reactions to temperature. Consequently, these values should not be interpreted independently as indicators of improved or reduced aging resistance. Instead, they should be evaluated together with the corresponding aromatization rate constants to provide a comprehensive description of the thermo-oxidative aging behavior of the investigated binders.

The results obtained by FTIR spectroscopy indicate that corn starch modifies the kinetics of oxidation reactions occurring within the bitumen matrix. However, the applied analytical approach does not provide direct evidence for the formation of new covalent bonds between starch and bitumen constituents. Considering the chemical structure of starch, which consists predominantly of amylose and amylopectin containing numerous hydroxyl groups, it is more likely that the modifier interacts physically with the polar fractions of bitumen through hydrogen bonding and secondary intermolecular interactions. These interactions may alter the colloidal organization of the binder, influence oxygen diffusion and consequently modify the rates of carbon oxidation, sulfur oxidation and aromatization observed in the present study. Therefore, the changes in FTIR indices should be interpreted primarily as alterations in the oxidation pathways of bitumen rather than as evidence of direct starch–bitumen chemical reactions.

### 3.5. MSCR Analysis

**Figure 7:**
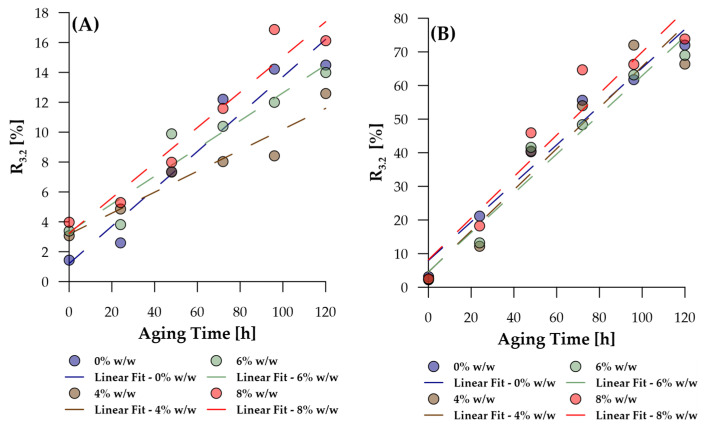
Changes in the R_3.2_ values of the investigated binders during aging at 100 °C (**A**) and 140 °C (**B**).

Bitumen aging not only affects the chemical composition of bitumen but also significantly alters its resistance to permanent deformation under repeated loading. These changes are reflected in the evolution of the binder’s viscoelastic properties, including increased stiffness and elastic response, which directly influence its rutting resistance and long-term pavement performance [57]. These changes are commonly reflected by an increase in the elastic recovery parameter (R) and a simultaneous decrease in the non-recoverable creep compliance (J_nr_), indicating progressive hardening and enhanced elastic response of the binder. Since both parameters are highly sensitive to transformations occurring during aging, their evolution can be effectively used to assess the kinetics of rheological property changes. Therefore, the determination of kinetic rate constants based on MSCR parameters provides valuable information on the aging susceptibility of bitumen binders and the influence of modifiers on the development of their deformation resistance [60]. In the present study, the changes in the recovery parameter measured at a stress level of 3.2 kPa (R_3.2_) were analyzed as a function of aging time. A systematic increase in R_3.2_ was observed during aging of both the reference and corn starch-modified bitumen binders, indicating a progressive enhancement of the elastic response of the binders associated with oxidation-induced stiffening and structural reorganization. The experimental data shown in Figure 7A,B were successfully described by a linear kinetic model (Equation (10)), yielding high coefficients of determination (R^2^ = 0.912–0.968). Consequently, the slope of the regression equation was adopted as the apparent rate constant of recovery development $k_{R_{3.2}}$.

(10) $$∆R_{3.2}=R_{3.2(t)}-R_{3.2(0)}=k_{R_{3.2}}\cdot t$$

Figure 7A shows that at 100 °C, all investigated bitumen binders exhibited a gradual increase in R_3.2_ with aging time at T = 100 °C, although substantial differences were observed among the investigated materials. The highest rate of recovery increase was determined for the reference binder, described by the equation R_3.2_ = 0.125∙t + 1.206 (R^2^ = 0.937), corresponding to a rate constant $k_{R_{3.2}}$ = 0.125%/h. A comparable value was obtained for SC8 bitumen $k_{R_{3.2}}\;=\;$0.1179∙t + 3.235 (R^2^ = 0.940), indicating a similar susceptibility to aging-induced enhancement of R_3.2_ value. Definitely, lower rates were observed for SC6 and SC4 bitumen binders, whose kinetic equations were R_3.2_ = 0.093∙t+3.329 (R^2^ = 0.918) and R_3.2_ = 0.0701∙t + 3.180 (R^2^ = 0.926), respectively. These behavior indicates that the increase in corn starch content generally reduced the rate of R_3.2_ increase, with SC4 bitumen exhibiting the greatest resistance to aging-related changes in viscoelastic behavior. The ranking of recovery growth rates during bitumen aging at T = 100 °C can therefore be expressed as Ref > SC8 > SC6 > SC4.

A markedly different behavior was observed at 140 °C, where significantly higher values of R_3.2_ were recorded for all binders. The reference binder exhibited a kinetic equation of R_3.2_ = 0.5731∙t + 7.955 (R^2^ = 0.968), corresponding to $k_{R_{3.2}}$ = 0.5731%/h. Similar $k_{R_{3.2}}\;$values were determined for SC4 and SC6 bitumen, characterized by the equations R_3.2_ = 0.6115∙t + 4.507 (R^2^ = 0.918) and R_3.2_ = 0.5831∙t + 4.616 (R^2^ = 0.957), respectively. The highest rate of R_3.2_ increase was observed for SC8, whose aging behavior was represented by the equation R_3.2_ = 0.618∙t + 8.171 (R^2^ = 0.919). The resulting rate constant exceeded those obtained for all other investigated bitumen binders, indicating the strongest sensitivity of SC8 to aging-induced increases in elastic recovery. The ranking of $k_{R_{3.2}}$ constants during bitumen aging at T = 140 °C can be expressed as SC8 ≈ SC4 > SC6 > Ref, although the differences between the binders were considerably smaller than those observed after aging at T = 100 °C.

Similarly to the structural changes observed using FTIR spectroscopy, the comparison of the $k_{R_{3.2}}$ values obtained at both temperatures reveals a strong temperature dependence of the R_3.2_ increase. Elevating the aging temperature from T = 100 °C to T = 140 °C resulted in an approximately 4.6-fold increase in $k_{R_{3.2}}$ for the reference binder, an 8.7-fold increase for SC4, a 6.3-fold increase for SC6 and a 5.2-fold increase for SC8. The increase in R_3.2_ values during aging simulation is consistent with the simultaneous increase in viscosity and oxidation indices observed for the investigated bitumen binders, indicating progressive hardening of the material and reduced susceptibility to permanent deformation. However, excessive increases in recovery may also indicate excessive stiffening of the binder, which could adversely affect low-temperature performance. Among the investigated bitumen binders, SC4 exhibited the lowest aging sensitivity at T = 100 °C, whereas SC8 showed the greatest increase in recovery at elevated temperature, suggesting that high starch contents promote the development of a more rigid and elastic structure during thermo-oxidative aging. The repeatability of the R_3_._2_ measurements was evaluated based on triplicate tests, and the corresponding standard deviations are summarized in Table 10.

Although these changes in J_nr3.2_ with aging time at T = 100 °C and T = 140 °C exhibited a hyperbolic trend, linear relationships obtained for ln(J_nr3.2_) = f(t) confirmed that the decrease in non-recoverable creep compliance follows first-order kinetics. This behavior indicates that the rate of rheological hardening is proportional to the amount of deformation-susceptible structures remaining in the binder and progressively decreases as aging proceeds. The linear relationships obtained for the plots of ln(J_nr3.2_) versus aging time exhibited high coefficients of determination (R^2^ = 0.919–0.976), confirming that the decrease in ln(J_nr3.2_) can be satisfactorily described by first-order reaction kinetics (Equation (11)). This behavior shows that the rate of rheological hardening is dependent not only on the current state of the binder structure but also on the concentration of reactive species participating in the aging process. Consequently, the determined rate constants $k_{{Jnr}_{3.2}}\;$may be interpreted as a quantitative measure of the rate at which the resistance of bitumen to permanent deformation develops during aging.

(11) $$ln(J_{nr})=\ln\left(J_{nr\left(0\right)}\right)-kt$$

**Figure 8:**
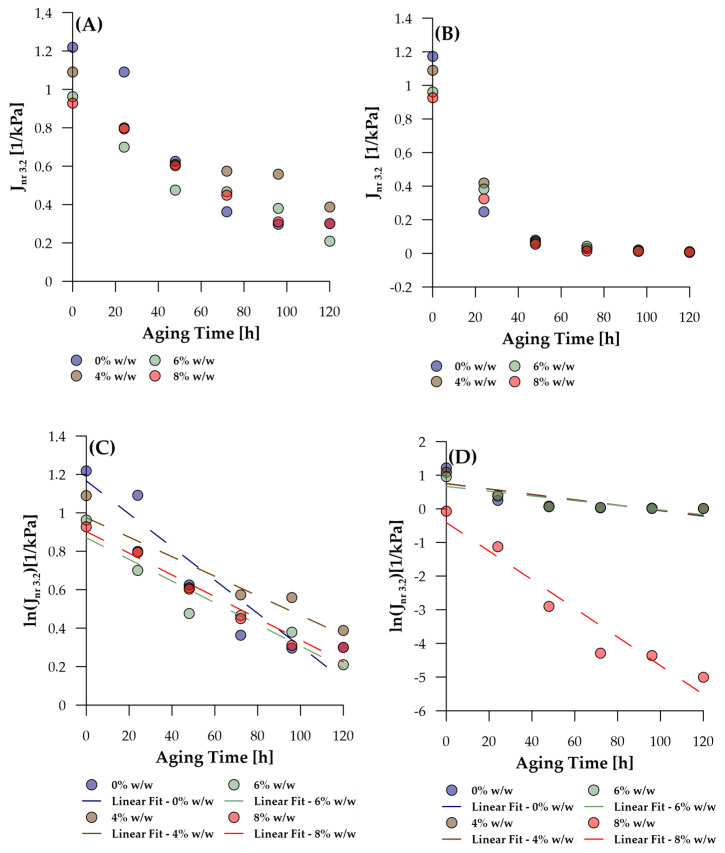
Changes in the Jnr_3.2_ values of the investigated binders during aging at 100 °C (**A**) and 140 °C (**B**) as well as changes in the ln(Jnr_3.2_) values of the investigated binders during aging at 100 °C (**C**) and 140 °C (**D**).

Figure 8C shows that kinetic equation ln(J_nr3.2_) = −0.0136∙t + 0.2152 (R^2^ = 0.9194) of neat bitumen, corresponding to a first-order rate constant of $k_{{Jnr}_{3.2}}$ = 0.0136 1/h. Slightly lower $k_{{Jnr}_{3.2}}\;$values were observed for SC6 and SC8 bitumen binders, for which the determined equations were ln(J_nr3.2_) = −0.0113∙t − 0.0622 (R^2^ = 0.9369) and ln(J_nr3.2_) = −0.0104∙t − 0.0391 (R^2^ = 0.9728), respectively. The lowest rate constant was determined for SC4, whose J_nr3.2_ values changes during aging was represented by the equation ln(J_nr3.2_) = −0.0075∙t − 0.0029 (R^2^ = 0.9210). Based on the obtained kinetic parameters, the susceptibility of the binders to aging-induced reduction in J_nr3.2_ followed the order: SC4 < SC8 < SC6 < Ref. These results suggest that corn starch modification of bitumen effectively retarded the development of permanent deformation resistance at moderate aging temperature, with the most pronounced protective effect observed for SC4 under the investigated aging conditions at 100 °C.

A substantially different aging behavior was observed at 140 °C (Figure 8B). The kinetic constants increased significantly for all investigated binders, demonstrating a strong acceleration of rheological aging processes under elevated thermal conditions. The reference binder was characterized by the equation ln(J_nr3.2_) = −0.0401∙t − 0.2789 (R^2^ = 0.9667), yielding $k_{{Jnr}_{3.2}}$ = 0.0401 1/h. Comparable values were obtained for SC6 and SC8, described by ln(J_nr3.2_) = −0.0408∙t − 0.1531 (R^2^ = 0.9560) and ln(J_nr3.2_) = −0.0443∙t − 0.2555 (R^2^ = 0.9256), respectively. The highest kinetic constant rate was determined for SC4, whose aging kinetics followed the equation ln(J_nr3.2_) = −0.0460∙t − 0.0274 (R^2^ = 0.9761). Thus, the rate of reduction in J_nr3.2_ during aging at T = 140 °C increased according to the sequence Ref < SC6 < SC8 < SC4. Unlike the behavior observed for aging at T = 100 °C, the SC4 binder became the most aging-sensitive material at an elevated temperature, exhibiting the fastest development of rutting resistance and the greatest rate of rheological hardening.

Comparison of the first-order kinetic constants obtained at both temperatures clearly demonstrates the strong temperature dependence of the mechanisms governing changes in J_nr3.2_. Increasing the aging temperature from 100 °C to 140 °C resulted in an approximately 2.9-fold increase in $k_{{Jnr}_{3.2}}$ for the reference bitumen binder, a 6.1-fold increase for SC4 bitumen, a 3.6-fold increase for SC6 bitumen and a 4.3-fold increase for SC8 bitumen. These results suggest that corn starch modification of road bitumen not only affects the magnitude of rheological changes during aging but also alters the temperature sensitivity of the underlying aging mechanisms. The exceptionally large increase observed for SC4 bitumen indicates that the protective effect of starch evident during aging at T=100 °C diminishes at higher temperatures, where accelerated oxidation and molecular restructuring processes dominate the aging response. Overall, the first-order kinetic analysis confirms that the evolution of permanent deformation resistance is governed by complex intermolecular interactions associated with thermo-oxidative aging and that starch content significantly influences the rate at which these structural transformations occur. All of the standard deviations of the Jnr_3_._2_ measurements are summarized in Table 11.

### 3.6. Mechanistic Interpretation of Chemical and Rheological Aging

**Scheme 1:**
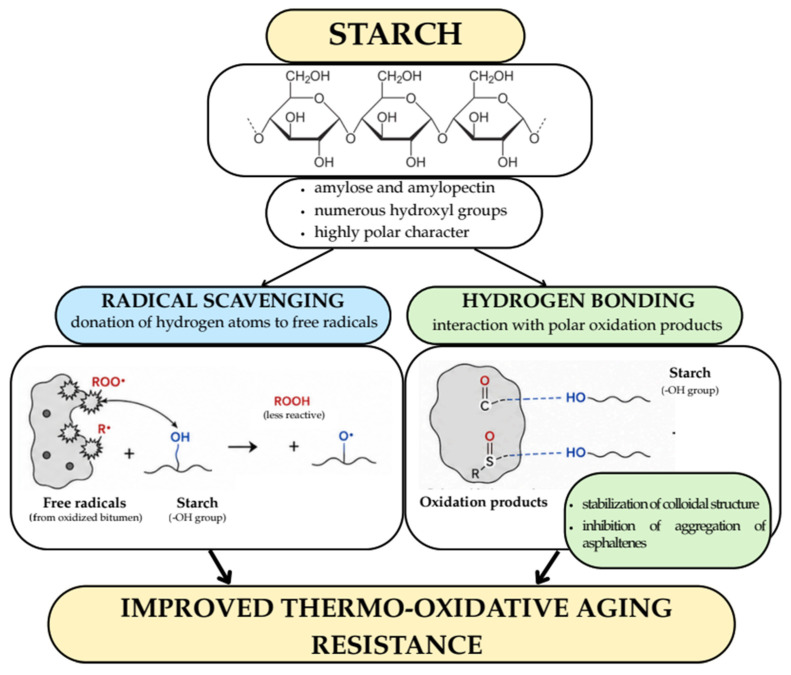
Proposed mechanism of the anti-aging action of corn starch in bitumen during thermo-oxidative aging.

Starch is a natural polysaccharide composed of amylose and amylopectin, whose glucose units contain numerous hydroxyl (-OH) groups. The presence of these functional groups imparts a highly polar character to starch molecules and enables the formation of hydrogen bonds with other oxygen-containing compounds [59,60]. Moreover, numerous studies on polysaccharides have demonstrated that hydroxyl-rich compounds may exhibit antioxidant activity by scavenging reactive oxygen species and free radicals. This mechanism is primarily based on the donation of hydrogen atoms to free radicals, thereby terminating the chain reactions responsible for the propagation of oxidation processes [61]. Therefore, it can be hypothesized that starch may interact in a similar manner within the bitumen matrix. The hydroxyl groups present in starch molecules could participate in scavenging free radicals generated during thermo-oxidative aging, thereby reducing the rate of carbonyl and sulfoxide group formation. Consequently, this mechanism could slow the increase in binder polarity, limit the transformation of lighter bitumen fractions into more polar oxidation products, and reduce the tendency of asphaltenes to aggregate. Furthermore, it cannot be excluded that the hydroxyl groups of starch may also interact with polar oxidation products through hydrogen-bond formation. Such interactions could partially stabilize the colloidal structure of bitumen and inhibit the further aggregation of the most polar constituents (Scheme 1). It should be emphasized that the proposed mechanism remains hypothetical and is inferred from the chemical characteristics of starch together with analogies to other bio-based modifiers used in asphalt technology. Its direct verification will require further studies employing advanced spectroscopic techniques and molecular modelling approaches to elucidate the molecular interactions between starch polysaccharides and asphalt binder constituents.

The combined analysis of the FTIR, dynamic viscosity and MSCR results provide a comprehensive description of the thermo-oxidative aging behavior of the investigated binders. The progressive formation of oxygen-containing functional groups, particularly carbonyl and sulfoxide species, increased the polarity of the bitumen matrix and promoted stronger intermolecular interactions among the polar fractions. These chemical transformations facilitated the association of asphaltene-like structures, leading to an increase in dynamic viscosity during aging. The rheological consequences of these structural changes were reflected in the MSCR parameters, where the progressive hardening of the binders resulted in increased elastic recovery (R_3.2_) and reduced non-recoverable creep compliance (Jnr_3.2_). Consequently, the FTIR indices, viscosity evolution and MSCR response should not be considered as independent aging indicators but rather as complementary descriptors of the same thermo-oxidative degradation process occurring within the bitumen. The consistency observed between the chemical and rheological indicators confirms that the proposed kinetic methodology enables comprehensive evaluation of aging behavior by linking molecular-scale oxidation processes with changes in engineering performance.

### 3.7. Economic Considerations and Practical Engineering Implications

In addition to the kinetic evaluation, a simplified economic analysis was performed to assess the effect of corn starch addition on binder production cost. The calculations were based on the market prices of paving-grade bitumen (2.38 PLN/kg), corn starch (3.31 PLN/kg) and the conventional SBS modifier (5.66 PLN/kg). The total production cost was estimated for one ton of modified binder containing 4, 6 and 8 wt.% modifier (Table 12). The results demonstrate that incorporating corn starch increased the production cost of bitumen from 2380 PLN/t for the reference binder to 2512.4 PLN/t, 2578.6 PLN/t and 2644.8 PLN/t for binders containing 4, 6 and 8 wt.% starch, respectively. This corresponds to cost increases of 5.6%, 8.3% and 11.1% relative to the unmodified binder. For comparison, the use of SBS at the same concentrations resulted in production costs of 2606.4 PLN/t, 2719.6 PLN/t and 2832.8 PLN/t, representing increases of 9.5%, 14.3% and 19.0%, respectively. Consequently, corn starch reduced the modification cost by approximately 94–188 PLN/t compared with SBS, depending on the modifier dosage. When these results are considered together with the aging kinetics presented in this study, the binder containing 4 wt.% corn starch appears to provide the most favorable balance between aging resistance and production cost. Higher starch dosages increased the production cost while not providing proportional improvements in aging resistance. Therefore, corn starch may be regarded not only as a renewable modifier but also as an economically attractive alternative to conventional polymer modifiers for selected bitumen applications.

The present results demonstrate that corn starch has considerable potential as a sustainable modifier for paving-grade bitumen. Within the investigated concentration range, the incorporation of corn starch influenced both the chemical aging mechanisms and the rheological evolution of the binder, indicating that bio-based modifiers may represent a promising alternative to conventional synthetic additives in selected pavement applications. From an engineering perspective, the improved control of thermo-oxidative aging may contribute to reducing the rate of binder hardening during asphalt production and its service life, thereby extending pavement durability and delaying the development of age-related distresses such as thermal cracking and fatigue damage. The kinetic methodology proposed in this study may also provide a useful tool for selecting modifier dosages based on both aging performance and temperature sensitivity. The economic assessment presented in this work further indicates that low corn starch contents, particularly 4 wt.%, provide a favorable balance between aging behavior and production cost within the investigated concentration range. Moreover, corn starch is a renewable, biodegradable and widely available agricultural product, which may contribute to reducing the environmental impact associated with petroleum-derived polymer modifiers. Nevertheless, several practical aspects require further investigation before large-scale implementation can be recommended. These include the storage stability of starch-modified binders during prolonged high-temperature storage, possible phase separation, the influence of moisture susceptibility, compatibility with different aggregate types, and the long-term field durability under traffic loading and environmental exposure. In addition, future studies should investigate the performance of starch-modified asphalt mixtures under realistic production conditions and evaluate their life-cycle environmental impacts.

## 4. Conclusions

This study presents a comprehensive kinetic evaluation of the thermo-oxidative aging of corn starch-modified bitumen under different aging temperatures. By combining rheological measurements, FTIR spectroscopy and Arrhenius analysis, it was possible to quantify the influence of both temperature and starch content on the rates of chemical transformations and rheological evolution occurring during road bitumen aging. Based on the obtained results, the following conclusions can be drawn:

- The proposed kinetic approach successfully quantified the aging process of both unmodified and corn starch-modified bitumen using complementary rheological (dynamic viscosity and MSCR) and chemical (FTIR) parameters. The high coefficients of determination confirmed that the selected kinetic models adequately describe the investigated aging processes.
- Aging temperature was identified as the dominant factor controlling the kinetics of bitumen degradation. Increasing the aging temperature from 100 °C to 140 °C significantly accelerated all analyzed chemical and rheological transformations, confirming the strong temperature dependence of the degradation process.
- Corn starch significantly modified the aging behavior of the investigated binders; however, its effectiveness depended on both aging temperature and modifier concentration. At 100 °C, the binder containing 4 wt.% corn starch exhibited the lowest rates of viscosity increase and rheological aging, whereas excessive starch content reduced the protective effect.
- FTIR analysis demonstrated that corn starch altered the kinetics of carbon oxidation, sulfur oxidation and aromatization reactions. Moreover, the modifier influenced the temperature sensitivity of these processes, as confirmed by the calculated activation energies.
- The Arrhenius analysis revealed that starch modification changes the apparent activation energies of the investigated aging reactions, indicating that the modifier affects not only the reaction rates but also the mechanisms governing degradation of bitumen.
- The MSCR results showed that the increase in the recovery parameter (R3.2) followed zero-order kinetics, whereas the reduction in non-recoverable creep compliance (Jnr3.2) was successfully described by a first-order kinetic model. These findings demonstrate that the evolution of bitumen rheological properties during aging can be quantitatively interpreted using reaction kinetics.
- Although no single binder exhibited the best performance for every investigated chemical and rheological indicator, the binder modified with 4 wt.% corn starch provided the most favorable overall balance between technological performance, chemical aging behavior, kinetic characteristics and production cost. Therefore, within the investigated concentration range (4–8 wt.% corn starch), this formulation exhibited the most favorable overall performance among the investigated binders. These findings should not be interpreted as evidence of a universal optimum modifier content, and further studies involving a broader range of starch concentrations are required to determine the optimum dosage more precisely. The proposed methodology, combining FTIR spectroscopy, rheological characterization and kinetic analysis, provides a comprehensive framework for evaluating the aging resistance of bio-modified bitumen and contributes to a better understanding of the mechanisms governing thermo-oxidative aging under different thermal conditions.
- The results demonstrate that the influence of corn starch cannot be evaluated using a single aging indicator, as the modifier affected different chemical and rheological aging processes to different extents depending on the aging temperature.

## Figures and Tables

**Table 1:** Basic properties of the reference 50/70 bitumen.

Property	Test Method	Unit of Measure	Result	Reference
Penetration at 25 °C	EN 1426	0.1 mm	66.3	[26]
Softening point	EN 1427	°C	49.7	[27]
Brookfield viscosity (160 °C)	ASTM D4402	mPa·s	261	[28]

**Table 2:** Designation and physicochemical properties of the investigated bitumen samples.

Sample Designation	Description	Penetration [0.1 mm]	Softening Point [°C]	Dynamic Viscosity [mPa∙s]
Ref	Unmodified 50/70 paving-grade bitumen	66.3	49.7	261
SC4	Bitumen modified with 4 wt.% corn starch	57.4	51.3	317
SC6	Bitumen modified with 6 wt.% corn starch	53.5	52.2	316
SC8	Bitumen modified with 8 wt.% corn starch	51.0	51.9	328

**Table 3:** Standard deviations of dynamic viscosity measurements obtained for the investigated binders during thermo-oxidative aging.

Aging Temperature [°C]		100				140				
Corn Starch Amount [% *w*/*w*]		0	4	6	8	0	4	6	8	SD [mPa∙s]
Aging Time [min]	0	13	17	16	15	11	10	9	12	
	24	14	18	18	15	18	17	16	22	
	48	16	18	20	18	30	27	30	34	
	72	20	19	20	18	44	44	32	44	
	96	21	21	20	21	50	50	51	56	
	120	22	23	24	25	76	49	49	80	

Standard deviations (SDs) were calculated from three independent viscosity measurements.

**Table 4:** Standard deviations of carbonyl index measurements obtained for the investigated binders during thermo-oxidative aging.

Aging Temperature [°C]		100				140				
Corn Starch Amount [% *w*/*w*]		0	4	6	8	0	4	6	8	SD [-]
Aging Time [min]	0	9 × 10^−6^	2 × 10^−5^	9 × 10^−6^	1 × 10^−5^	1 × 10^−5^	1 × 10^−5^	2 × 10^−5^	2 × 10^−6^	
	24	9 × 10^−6^	4 × 10^−5^	4 × 10^−5^	2 × 10^−5^	8 × 10^−5^	4 × 10^−5^	2 × 10^−5^	1 × 10^−5^	
	48	1 × 10^−5^	1 × 10^−4^	4 × 10^−5^	2 × 10^−5^	2 × 10^−4^	2 × 10^−4^	3 × 10^−5^	4 × 10^−5^	
	72	3 × 10^−5^	2 × 10^−4^	7 × 10^−5^	4 × 10^−5^	3 × 10^−4^	2 × 10^−4^	8 × 10^−5^	9 × 10^−5^	
	96	3 × 10^−5^	2 × 10^−4^	7 × 10^−5^	5 × 10^−5^	2 × 10^−4^	3 × 10^−4^	1 × 10^−4^	1 × 10^−4^	
	120	5 × 10^−5^	2 × 10^−4^	7 × 10^−5^	7 × 10^−5^	4 × 10^−4^	2 × 10^−4^	1 × 10^−4^	1 × 10^−4^	

Standard deviations (SDs) were calculated from three independent viscosity measurements.

**Table 5:** Kinetic parameters of carbonyl group formation during thermo-oxidative aging of bitumen modified with different corn starch contents.

Corn Starch Content [% *w*/*w*]	$k_{I_{C=O}}$ (T = 100 °C) [1/h]	$k_{I_{C=O}}$ (T = 140 °C) [1/h]	Ea [kJ/mol]
0	9.00 × 10^−6^	7.00 × 10^−5^	67.6
4	4.00 × 10^−5^	5.00 × 10^−5^	17.1
6	1.00 × 10^−5^	2.00 × 10^−5^	22.1
8	1.00 × 10^−5^	3.00 × 10^−5^	35.4

**Table 6:** Standard deviations of sulphoxide index measurements obtained for the investigated binders during thermo-oxidative aging.

Aging temperature [°C]		100				140				
Corn starch amount [% *w*/*w*]		0	4	6	8	0	4	6	8	SD [-]
Aging time [min]	0	0.00028	0.00046	0.00029	0.00073	0.00029	0.00029	0.00033	0.00086	
	24	0.00035	0.00065	0.00047	0.00080	0.00066	0.00066	0.00033	0.00056	
	48	0.00038	0.00063	0.0005	0.00102	0.00080	0.00080	0.00034	0.00065	
	72	0.00044	0.00066	0.00067	0.00087	0.00075	0.00075	0.00040	0.00090	
	96	0.00046	0.00086	0.00081	0.00103	0.00090	0.00090	0.00075	0.00172	
	120	0.00087	0.00101	0.00070	0.00116	0.00119	0.00119	0.00110	0.00170	

Standard deviations (SDs) were calculated from three independent viscosity measurements.

**Table 7:** Kinetic parameters of sulfoxide group formation during thermo-oxidative aging of bitumen modified with different corn starch contents.

Corn Starch Content [% *w*/*w*]	$k_{I_{S=O}}$ (T = 100 °C) [1/h]	$k_{I_{S=O}}$ (T = 140 °C) [1/h]	Ea [kJ/mol]
0	9.87 × 10^−5^	1.17 × 10^−4^	5.33
4	1.04 × 10^−4^	1.72 × 10^−4^	16.03
6	9.77 × 10^−5^	1.53 × 10^−4^	14.42
8	8.60 × 10^−5^	2.69 × 10^−4^	36.53

**Table 8:** Standard deviations of aromaticity index measurements obtained for the investigated binders during thermo-oxidative aging.

Aging Temperature [°C]		100				140				
Corn Starch Amount [% *w*/*w*]		0	4	6	8	0	4	6	8	SD [-]
Aging Time [min]	0	9 × 10^−6^	2 × 10^−5^	1 × 10^−5^	1 × 10^−5^	9 × 10^−6^	2 × 10^−5^	1 × 10^−5^	1 × 10^−5^	
	24	1 × 10^−5^	3 × 10^−5^	3 × 10^−5^	1 × 10^−5^	8 × 10^−5^	4 × 10^−5^	1 × 10^−5^	1 × 10^−5^	
	48	2 × 10^−5^	1 × 10^−4^	3 × 10^−5^	2 × 10^−5^	3 × 10^−4^	2 × 10^−4^	3 × 10^−5^	5 × 10^−5^	
	72	4 × 10^−5^	2 × 10^−4^	6 × 10^−5^	4 × 10^−5^	3 × 10^−4^	2 × 10^−4^	8 × 10^−5^	1 × 10^−4^	
	96	4 × 10^−5^	2 × 10^−4^	7 × 10^−5^	5 × 10^−5^	3 × 10^−4^	2 × 10^−4^	1 × 10^−4^	1 × 10^−4^	
	120	5 × 10^−5^	2 × 10^−4^	7 × 10^−5^	5 × 10^−5^	4 × 10^−4^	3 × 10^−4^	1 × 10^−4^	1 × 10^−4^	

Standard deviations (SDs) were calculated from three independent viscosity measurements.

**Table 9:** Kinetic parameters of the aromatization process during thermo-oxidative aging of bitumen modified with different corn starch contents.

Corn Starch Content [% *w*/*w*]	$k_{I_{\mathrm{aromatization}}}$ (T = 100 °C) [1/h]	$k_{I_{\mathrm{aromatization}}}$ (T = 140 °C) [1/h]	Ea [kJ/mol]
0	7.54 × 10^−5^	1.03 × 10^−4^	4.75
4	9.68 × 10^−5^	1.90 × 10^−4^	20.80
6	3.00 × 10^−5^	2.00 × 10^−4^	48.42
8	1.00 × 10^−4^	1.04 × 10^−4^	1.31

**Table 10:** Standard deviations of R3.2 measurements obtained for the investigated binders during thermo-oxidative aging.

Aging Temperature [°C]		100				140				
Corn Starch Amount [% *w*/*w*]		0	4	6	8	0	4	6	8	SD [%]
Aging Time [min]	0	0.059	0.115	0.139	0.179	0.059	0.103	0.109	0.106	
	24	0.120	0.194	0.170	0.243	0.970	0.462	0.544	0.827	
	48	0.351	0.295	0.447	0.354	1.895	1.794	1.904	2.160	
	72	0.510	0.351	0.452	0.474	2.410	2.557	2.035	2.604	
	96	0.568	0.409	0.562	0.732	2.828	3.333	2.766	2.853	
	120	0.643	0.475	0.542	0.650	2.708	2.948	3.083	2.870	

Standard deviations (SDs) were calculated from three independent viscosity measurements.

**Table 11:** Standard deviations of Jnr3.2 measurements obtained for the investigated binders during thermo-oxidative aging.

Aging Temperature [°C]		100				140				
Corn Starch Amount [% *w*/*w*]		0	4	6	8	0	4	6	8	SD [kPa^−1^]
Aging Time [min]	0	0.057	0.047	0.040	0.039	0.057	0.043	0.041	0.038	
	24	0.046	0.034	0.034	0.037	0.010	0.016	0.018	0.014	
	48	0.028	0.025	0.021	0.025	0.002	0.002	0.0029	0.002	
	72	0.017	0.021	0.019	0.019	0.001	0.001	0.0019	0.0005	
	96	0.012	0.025	0.016	0.013	0.0008	0.0004	0.0005	0.0006	
	120	0.013	0.016	0.008	0.012	0.0003	0.0002	0.0004	0.0002	

Standard deviations (SDs) were calculated from three independent viscosity measurements.

**Table 12:** Estimated production cost of bitumen modified with corn starch and SBS at different modifier contents.

Bitumen Modifier	4% *w*/*w*	6% *w*/*w*	8% *w*/*w*
Corn starch	2512.40	2578.60	2644.80
SBS copolymer	2606.40	2719.60	2832.80
Difference (PLN/t)	94.00	141.00	188.00

## Data Availability

The original contributions presented in this study are included in the article. Further inquiries can be directed to the corresponding author.

## Abbreviations

The following abbreviations are used in this manuscript:

ATR	Attenuated Total Reflectance
ASTM	American Society for Testing and Materials
DSR	Dynamic Shear Rheometer
Ea	Apparent activation energy
FTIR	Fourier Transform Infrared Spectroscopy
MSCR	Multiple Stress Creep Recovery
Ref	Unmodified reference bitumen
SC4	Bitumen modified with 4 wt.% corn starch
SC6	Bitumen modified with 6 wt.% corn starch
SC8	Bitumen modified with 8 wt.% corn starch
η	Dynamic viscosity
Δη	Change in dynamic viscosity during bitumen aging
η(t)	Dynamic viscosity after *t* hours of aging
η(0)	Initial dynamic viscosity before aging
kη	Apparent rate constant of viscosity increase
I_C=O_	Carbonyl index
ΔI_C=O_	Change of carbonyl index during bitumen aging
I_C=O(t)_	Carbonyl index after *t* hours of aging
I_C=O(0)_	Initial carbonyl index before aging
$k_{I_{C=O}}$	Apparent rate constant of carbonyl formation
I_S=O_	Sulfoxide index
ΔI_S=O_	Change in sulfoxide index during bitumen aging
I_S=O(t)_	Sulfoxide index after *t* hours of aging
I_S=O(0)_	Initial sulfoxide index before aging
k_IS=O_	Apparent rate constant of sulfoxide formation
I_C=C_	Aromaticity index
ΔI_aromaticity_	Change in aromaticity index during bitumen aging
I_aromaticity(t)_	Aromaticity index after *t* hours of aging
I_aromaticity(0)_	Initial aromaticity index before aging
k_Iaromatization_	Apparent rate constant of the aromatization process
R	Percent recovery
ΔR_3.2_	Change in percent recovery during bitumen aging at a stress level of 3.2 kPa
R_3.2(t)_	Percent recovery after *t* hours of aging at a stress level of 3.2 kPa
R_3.2(0)_	Initial percent recovery before aging at a stress level of 3.2 kPa
k_R3.2_	Apparent rate constant of changes in percent recovery at a stress level of 3.2 kPa
J_nr_	Non-recoverable creep compliance
J_nr(0)_	Initial non-recoverable creep compliance before aging
ΔJ_nr3.2_	Change in non-recoverable creep compliance during bitumen aging at a stress level of 3.2 kPa
J_nr3.2(t)_	Non-recoverable creep compliance after *t* hours of aging at a stress level of 3.2 kPa
J_nr3.2(0)_	Initial non-recoverable creep compliance before aging at a stress level of 3.2 kPa
k_Jnr3.2_	Apparent rate constant of changes in non-recoverable creep compliance at a stress level of 3.2 kPa
ε_nr_	Non-recoverable strain
ε_max_	Maximum strain during the creep phase
σ	Applied creep stress
A_1700_	Integrated absorbance of the carbonyl band at 1700 cm^−1^
A_1030_	Integrated absorbance of the sulfoxide band at 1030 cm^−1^
A_1600_	Integrated absorbance of the aromatic C=C band at 1600 cm^−1^
ΣA	Sum of integrated absorbance values used for FTIR index normalization
R^2^	Coefficient of determination
t	Aging time
